# MIBG Scintigraphy and Arrhythmic Risk in Myocarditis

**DOI:** 10.3390/biomedicines13081981

**Published:** 2025-08-15

**Authors:** Maria Lo Monaco, Margherita Licastro, Matteo Nardin, Rocco Mollace, Flavia Nicoli, Alessandro Nudi, Giuseppe Medolago, Erika Bertella

**Affiliations:** 1 Humanitas Gavazzeni, Via Mauro Gavazzeni, 21, 24125 Bergamo, BG, Italy; margherita.licastro@gavazzeni.it (M.L.); rocco.mollace@gavazzeni.it (R.M.); flavia.nicoli@gavazzeni.it (F.N.); alessandro.nudi@gavazzeni.it (A.N.); giuseppe.medolago@gavazzeni.it (G.M.); erika.bertella@gavazzeni.it (E.B.); 2 IRCCS Humanitas Research Hospital, Via Alessandro Manzoni, 56, 20089 Rozzano, MI, Italy

**Keywords:** myocarditis, cardiac MRI, ^123^I-MIBG scintigraphy, arrhythmic risk, sympathetic nervous system, late gadolinium enhancement, ventricular arrhythmias

## Abstract

**Background:** The widespread use of cardiac magnetic resonance imaging (MRI) in clinical practice has enabled the identification of numerous patients with evident damage from previous myocarditis, whether known or unknown. For years, myocardial fibrosis has been a topic of interest due to its established correlation with arrhythmic events in various clinical settings, including ischemic heart disease, dilated cardiomyopathy, and hypertrophic cardiomyopathy. MIBG scintigraphy is a method widely used in patients who are candidates for defibrillator implantation or have experienced heart failure. This examination evaluates the sympathetic innervation of the myocardium. **Objective:** To assess the real arrhythmogenic risk of non-ischemic scars identified in symptomatic or asymptomatic patients through the use of MIBG. **Methods:** Patients were retrospectively selected based on the presence of non-ischemic myocardial fibrosis detected by cardiac MRI, consistent with a myocarditis outcome (even in the absence of a clear history of myocarditis). These patients underwent myocardial scintigraphy with MIBG using a tomographic technique. **Results:** A total of 50 patients (41 males, mean age 51 ± 16 years) who underwent MRI from 2019 to June 2024 were selected. The primary indication for MRI was ventricular ectopic extrasystoles detected on Holter ECG (*n* = 12, 54%), while five patients underwent MRI following a known acute infectious event (23%, including three cases of COVID-19 infection). All symptomatic patients presented with chest pain in the acute phase, accompanied by elevated hsTNI levels (mean value: 437 pg/mL). The MRI findings showed normal ventricular volumes (LV: 80 mL/m^2^, RV: 81 mL/m^2^) and normal ejection fractions (56% and 53%, respectively). The mean native T1 mapping value was 1013 ms (normal range: 950–1050). T2 mapping values were altered in the 5 patients who underwent MRI during the acute phase (mean value: 57 ms), without segmentation. Additionally, three patients had non-tamponade pericardial effusion. All patients exhibited LGE (nine subepicardial, seven midwall, six patchy). All patients underwent myocardial scintigraphy with MIBG at least 6 months after the acute event, with only one case yielding a positive result. This patient, a 57-year-old male, had the most severe clinical presentation, including more than 65,000 premature ventricular beats (PVBs) and multiple episodes of paroxysmal supraventricular tachycardia (PSVT) recorded on Holter ECG. MRI findings showed severe left ventricular dysfunction, a slightly dilated LV, and midwall LGE at the septum, coinciding with hypokinetic areas. **Conclusions:** MIBG scintigraphy could be a useful tool in assessing arrhythmic risk in patients with previous myocarditis. It could help reduce the clinical burden of incidental findings of non-ischemic LGE, which does not appear to be independently associated with an increased risk profile.

## 1. Introduction

Myocarditis is an inflammatory condition affecting the myocardium, involving myocytes and interstitial tissue, leading to cellular edema, necrosis, and fibrosis. It can result from various causes, but it typically follows an acute viral infection or occurs as an autoimmune response to viral exposure [1].

Despite the established role of the sympathetic nervous system (SNS) [2] in cardiac arrhythmias and the importance of myocarditis, a significant research gap exists: there is a lack of consistent studies correlating prior myocarditis with arrhythmic risk. While ^123^I-MIBG myocardial scintigraphy shows promise in assessing cardiac SNS integrity for identifying patients at risk of malignant arrhythmias [3,4,5,6], and cardiac magnetic resonance imaging (MRI) excels at detecting structural myocardial damage (both acute and chronic), it remains unclear how combining or comparing these two non-invasive techniques can improve arrhythmic risk stratification in patients with a history of myocarditis. The current “gold standard,” endomyocardial biopsy, is invasive, and its clinical use remains controversial [7,8].

Cardiac magnetic resonance imaging (MRI) is a diagnostic technique based on the application of a high-intensity magnetic field. Compared to other visualization methods, it is characterized by minimal invasiveness and the absence of ionizing radiation [9].

**Figure 1:**
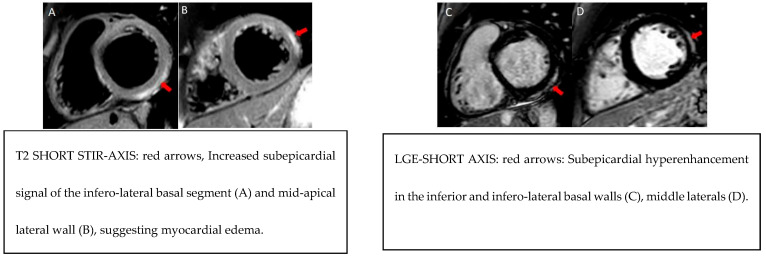
Example MRI findings.

We hypothesize that integrating data from ^123^I-MIBG scintigraphy (for SNS dysfunction assessment) and cardiac MRI with T1/T2 mapping and Late Gadolinium Enhancement (LGE) (for characterizing myocardial damage, edema, and fibrosis) [10,11] can provide a more comprehensive and accurate assessment of arrhythmic risk in patients with prior myocarditis (Figure 1). This combined approach could overcome the limitations of individual methods and invasive procedures. Specifically, we propose that altered ^123^I-MIBG signals, coupled with MRI-detected edema and fibrosis, may serve as a more robust indicator of electrical instability and susceptibility to ventricular arrhythmias.

## 2. Materials and Methods

### 2.1. Study Population

The study sample included 50 patients, selected retrospectively based on the presence of LGE (with a non-ischemic pattern) on MRI images. The MRI study protocol was performed using a 1.5-T whole-body scanner (MAGNETOM Aera; Siemens Healthcare, Erlangen, Germany) with a phased-array body coil. The sample selection window covered MRI reports from 2019 to June 2024. Patients’ voluntary participation was documented via signed informed consent.

More than 82% of the examined subjects were male adults, with an average age of 51 years.

The MRI examination was performed on an outpatient basis for indications of any type of ventricular arrhythmia. Based on the reports examined, 13 patients had arrhythmias detected during exercise testing (BEV_stress) and 9 had a 24 h Dynamic Holter ECG with >2000 BEVs. However, 10% of patients experienced episodes of non-sustained ventricular tachycardia (NSVT). None of the selected patients had ventricular fibrillation or prolonged ventricular tachycardia. Another indication for MRI was an acute event. In these cases, the patients were admitted to the Cardiology Department for a variable period (from a few days to a couple of weeks) due to specific cardiac symptoms such as dyspnea, chest pain, suspected syncope, troponin elevation, or non-specific flu-like symptoms such as fever, vomiting, and diarrhea. In 18% of the acute cases found, the discharge diagnosis was perimyocarditis, a condition that suggests secondary involvement of the pericardium with possible moderate pericardial effusion.

In the acute cases studied, hospitalization was linked to a viral infection, likely caused by the SARS-CoV-2 virus. The incidence of myocarditis was previously between 1 and 10 cases per 100,000 people per year. However, since the advent of COVID-19, the incidence is estimated at 146 cases per 100,000. Recent studies show that adults who develop COVID-19-related myocarditis tend to have worse outcomes than those with myocarditis unrelated to SARS-CoV-2, including a higher risk of sudden death [12].

### 2.2. Data Collection and Data Analysis

All subjects underwent MRI, and among the MRI parameters reported, the average left ventricular end-diastolic volume per body surface area (LV_EDV/m^2^) was 85.6 mL/m^2^ (range: 47–122 mL/m^2^) (Table 1). Less than 20% of patients (10 out of 50) exhibited increased left ventricular size at end-diastole. Approximately 16% (8 patients) showed moderate left ventricular hypertrophy, with increased septal or concentric wall thickness up to 13 mm.

The ejection fraction is a key clinical parameter for quantifying heart pump function and myocardial contractility. In the studied sample, the average left ventricular ejection fraction (LV_EF) was 55.6%, with a range of 26–73%. A reduction in this parameter was observed in 35% of patients (8 cases), but only one case showed severe left ventricular dysfunction, confirmed by Doppler echocardiography findings of left ventricular dilation, “sphericization,” septal thinning (akinetic in the mid-basal portion), and diffuse hypokinesia.

Nearly 30% (*n* = 15) of patients exhibited left ventricular wall motion abnormalities (WM_abnormalities). This parameter is relevant for risk stratification when kinetic alterations align with myocardial damage, as identified by LGE on MRI. This concordance was verified in 100% of the 9 described cases. Pericardial effusion, secondary to pericardial inflammation, was observed in 3 of the 9 acute perimyocarditis cases.

In 11 out of 44 MRI reports (25%), a native T1 mapping signal was detected in areas corresponding to myocardial fibrosis (native_T1_mapping_abnormality). Of these, 66% (*n* = 4) underwent the examination a few days after the acute symptomatic event. Unfortunately, no information is available on the remaining four cases due to the presence of “breath artifacts.” On the other hand, post-contrast T1 mapping is primarily used to calculate the extracellular volume (ECV) fraction. Standard gadolinium-based contrast agents distribute in the extracellular space and reduce the T1 relaxation time of the myocardium in proportion to the local gadolinium concentration. Consequently, areas of fibrosis and scarring exhibit reduced T1 relaxation times after contrast administration. The patient’s ECV (interstitial and extracellular matrix volume) value is estimated as follows:

$$ECV=\left(1-haematocrit\right)\frac{\frac{1}{post\;contrast\;T1\;myo}-\;\frac{1}{native\;T1\;myo}}{\frac{1}{post\;contrast\;T1\;blood}-\frac{1}{native\;T1\;blood}}$$

ECV serves as an indicator of myocardial tissue remodeling following damage. Normal ECV values of 25.3 ± 3.5% [1.5 T] have been reported in healthy individuals [13]. An increase in ECV is generally attributed to excessive collagen deposition. In the study sample, this parameter increased in 4 out of 50 cases, representing the number of ECV maps available for clinical interpretation. As anticipated, selected patients exhibited areas of late gadolinium enhancement (LGE) (LV_LGE), which were appreciable in cardiac MRI images, particularly in T1-weighted sequences following gadolinium contrast administration [13]. The LGE distribution was subepicardial (38%, *n* = 19), midwall (42%, *n* = 21), or patchy (20%, *n* = 10). No patient exhibited LGE at the subendocardial level.

The extent of myocardial scarring (%LGE) is expressed as the ratio of fibrotic myocardial mass (gr_LGE) to total myocardial mass. This parameter quantifies the severity of damage and the level of cardiac impairment caused by myocarditis, ranging from 1% to 25%. The average value was <7% (6.2%).

The number of affected myocardial segments ranged from 0 to 8, with an average of 2.34. The well-known “17-segment model” was used to locate the LGE area. Additionally, each segment’s fibrotic area was qualitatively assessed. In this study, segmental involvement was classified as follows: (1) <25%, (2) 25–50%, (3) 50–75%, and (4) >75%. In 32% of cases, the damage affected more than 50% of the segment.

The following analysis includes a total of 18 patients, as the remaining images were not assessable due to artifacts and poor image quality. The most commonly affected segments were the basal inferolateral (50%, *n* = 28), middle inferolateral (55.5%, *n* = 17), middle anterolateral, and basal inferior (*n* = 10). Occasional involvement of the basal anterolateral (16.6%, *n* = 9) and apical lateral (22.2%, *n* = 8) portions was noted. However, no patient exhibited LGE along the basal and apical anterior wall or at the “true apex.”

### 2.3. Scintigraphy Data with ^123^I-MIBG

All patients underwent myocardial scintigraphy with a ^123^I-MIBG tracer.

The administered dose ranged from 3 to 5 mCi (equivalent to 111–185 MBq). The radiopharmaceutical was injected at rest, 15 min before acquiring the early static planar chest image. The late phase of the protocol was conducted approximately 3–4 h later. Patient preparation involved discontinuation of medications that might interfere with ^123^I-MIBG absorption, such as antidepressants and antipsychotics. Given the radioactive nature of the procedure, the patients were advised to avoid contact with children or pregnant women for 24–48 h post-injection (the period during which the body naturally eliminates the compound).

Image reprocessing (early and late) involved graphical representation of regions of interest (ROI). It was essential to cover the entire myocardium while carefully excluding adjacent lung and liver activity. The mediastinal region (rectangular ROI) was positioned centrally in the mediastinum, below the thyroid. “Thyroid blockade” was implemented to reduce thyroid radionuclide uptake by administering Lugol’s solution (aqueous iodine solution) beforehand.

Three primary quantitative indices were analyzed: the early heart-to-mediastinum (H/M) ratio, which reflects receptor density and myocardial presynaptic nerve uptake capacity; the late H/M ratio, indicating the functional efficacy of the adrenergic system; the myocardial washout rate (WOR), calculated from early and late images and expressed as a percentage, representing the degree of tracer clearance. A decreased H/M ratio indicates impaired myocardial adrenergic innervation, whereas an increased WOR suggests inefficiency in physiological neurotransmitter (norepinephrine) recirculation.

WOR is also a valid indicator of adrenergic dysfunction. A study on 73 patients with chronic heart failure showed that those with abnormal WOR (>27%) had a significantly higher incidence of sudden cardiac death than those with normal WOR [14].

These three semi-quantitative parameters facilitate the assessment of sympathetic nervous system (adrenergic) impairment in the myocardium and, consequently, the patient’s risk of malignant arrhythmic events. Scintigraphic examination was conducted using a “single rest” protocol on a single day, employing planar imaging in the early anterior projection. During acquisition, the patients were positioned supine on the Gamma Camera table with arms raised above their heads to minimize interference from anatomical structures in the chest and underlying liver activity.

This study considered the following normal values: early H/M ratio (EHMR) >1.60, late H/M ratio (LHMR) > 1.60, and WOR <30%. The average EHMR was 1.8 (max = 2.28, min = 1.35); the average LHMR was 1.88 (max = 2.77, min = 1.35); and the average WOR was 22.4 (max = 49.88, min = 5.5).

Based on these parameters, the arrhythmogenic risk level was estimated. A “low” risk profile was identified in 92% of cases. However, 4 out of 50 patients exhibited an intermediate/high-risk profile (significantly low EHMR and LHMR, and high WOR). One notable case involved a 57-year-old woman with severe clinical and MRI findings: over 65,000 premature ventricular contractions (PVCs) recorded on a 24 h Holter ECG, severe left ventricular dysfunction confirmed by MRI, and a diagnosis of dilated cardiomyopathy. Consequently, she underwent primary prevention CRT-D implantation.

### 2.4. Follow-Up Data

A few months after the scintigraphic examination, the selected patients were contacted by telephone for a brief update on their current health status. The median follow-up period was six months.

The protocol of the present study includes a follow-up phase, during which the last contact with the patient is focused on completing a simple questionnaire investigating the possible onset of symptomatic arrhythmias. Follow-up is also useful for collecting data related to the periodic monitoring of malignant arrhythmias using a 24 h Holter ECG. During this phase, the patients were asked to authorize the use of data from their cardiological examinations performed after the scintigraphy.

**Figure 2:**
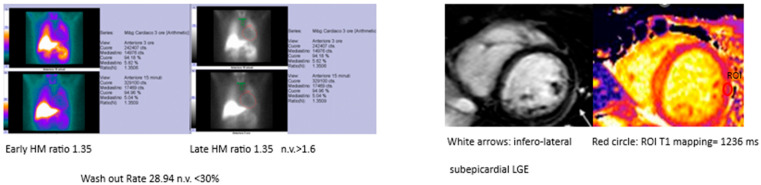
Example MIBG and MRI findings in the same patient.

From the data collected, it emerged that 88% (*n* = 44) of patients did not experience symptomatic arrhythmias, heart palpitations, or episodes of irregular heartbeat in recent months. The remaining 12% (*n* = 6) reported the following:

- In total, 2 patients experienced fewer than five episodes of irregular heartbeat in the last three months, each lasting a few seconds, occurring at rest, and not associated with additional symptoms.
- In total, 1 patient reported brief but very frequent episodes of irregular heartbeat (over 30 in three months), occurring at rest or during the night and accompanied by a sensation of shortness of breath.
- In total, 1 patient experienced rare episodes (fewer than five in three months) of an altered heartbeat at the beginning of exertion, lasting approximately five minutes.
- In total, 1 patient reported a single nocturnal tachycardia episode.
- In total, 1 patient underwent a cardioversion protocol following prolonged atrial fibrillation (12 h).
- At follow-up, a 24 h Holter ECG report revealed one case of BEV >2000 and non-sustained ventricular tachycardia (NSVT), consistent with findings documented during the first phase of the protocol (Figure 2). These asymptomatic arrhythmic episodes are not of new onset. However, it should be noted that, to date, only six patients’ 24 h Holter ECG reports are available. For this reason, further analyses would not be supported by sufficient clinical data.

For the present statistical analysis, the training set is shown in the table below (Table 2) and corresponds to a subset of the dataset related to the sample of 50 patients described in the previous chapter. The independent variables selected for model construction include only personal factors (age and sex) and the semi-quantitative parameters obtained from myocardial scintigraphy with ^123^I-MIBG (EHMR, LHMR, WOR), previously discussed. The dependent variable is the level of arrhythmic risk, as estimated by the Nuclear Physician, based on the degree of impairment of myocardial adrenergic innervation. This is a dichotomous variable with only two categories (0 = low risk; 1 = increased risk).

This estimate results from a simple decision-making process, primarily based on comparing the EHMR, LHMR, and WOR values obtained from the scintigraphic protocol with the normal value ranges reported in the literature. For the present study, the considered normal values are as follows, as mentioned before: EHMR > 1.60, LHMR > 1.60, WOR < 30%.

## 3. Discussion

Acute viral myocarditis and acute pericarditis are self-limiting conditions that generally follow a benign course and may not always present with symptoms prompting medical evaluation. However, ventricular arrhythmia is common in viral myocarditis. Myocarditis is believed to account for a significant proportion of sudden cardiac deaths (SCDs) in young individuals without prior structural heart disease. The proportion of SCDs attributed to myocarditis at autopsy varies by age, accounting for approximately 2% of cases in infants (0–2 years), 5% in children (3–18 years), and between 10% and 20% in young adults (19–44 years) [15,16,17,18].

Identifying acute myocarditis, with or without pericarditis, is therefore crucial. However, therapeutic interventions remain limited and largely nonspecific. Identifying patients at the highest risk of life-threatening arrhythmias is critical to reducing mortality [19].

The arrhythmic risk profile of myocarditis patients is estimated based on specific semi-quantitative variables derived from myocardial scintigraphy images using the ^123^I-MIBG tracer. This approach allows each patient to be assigned a hypothetical risk level, reflecting their likelihood of developing malignant arrhythmias in the future. For example, a study conducted on 170 patients with advanced chronic heart failure (CHF) and an implantable cardioverter defibrillator (ICD) found that a decreased late heart/mediastinum (H/M) ratio (threshold = 1.54) was associated with a higher incidence of ICD discharges, with a positive predictive value of 71% [6,20].

In other two additional prospective studies, ADMIRE-HF and ADMIRE-HFX, conducted on 961 patients with New York Heart Association (NYHA) functional class II/III heart failure (HF) and a left ventricular ejection fraction (LVEF) ≤ 35%, participants underwent ^123^I-MIBG myocardial imaging, which quantified sympathetic neuronal integrity using the H/M uptake ratio on 4 h delayed planar images, as well as myocardial perfusion imaging. They were then followed for up to two years. Time to first occurrence of NYHA functional class progression, potentially life-threatening arrhythmic events, or cardiac death was analyzed in relation to H/M (either as a continuous variable or relative to an estimated lower limit of normal [1.60] using Cox proportional hazards models. ADMIRE-HF provided prospective validation of the independent prognostic value of ^123^I-MIBG scintigraphy in the assessment of HF patients [21,22].

This study is an observational analysis focused on the stratification of arrhythmic risk in patients with a history of myocarditis. Cardiac magnetic resonance imaging (MRI) of the selected patients revealed areas of late gadolinium enhancement (LGE) in the left ventricle, indicative of scarring and myocyte necrosis. In current clinical practice, such patients are considered to be at high risk for arrhythmias. Consequently, current guidelines recommend specific pharmacological therapies and restrictions on high-intensity physical exertion.

Through the use of ^123^I-MIBG cardiac scintigraphy, it is possible to assess an individual’s actual predisposition to life-threatening arrhythmias. The radiopharmaceutical ^123^I-MIBG, administered for imaging, measures myocardial tissue excitability and highlights the severity of damage to adrenergic receptor innervation. This enables the identification of patients with an increased susceptibility to malignant arrhythmias, providing a reliable method for risk stratification.

When used alongside MRI, this procedure enhances arrhythmic risk assessment in patients with prior myocarditis. It reduces the clinical burden associated with the incidental finding of non-ischemic LGE, which alone is an insufficient criterion for determining an individual’s risk profile. Understanding a patient’s level of risk for adverse events is essential for implementing targeted management and treatment strategies. The results obtained are clearly insufficient to demonstrate the reliability of the model.

## 4. Study Limitations

Some limitations have to be acknowledged in this work. It was a single-center study conducted in a population of extremely small size with a short follow-up. The single-center design represents a weakness.

Furthermore, the heterogeneity of the study population inevitably impacts the results.

For this reason, it is advisable to test the model on a sufficiently large sample of patients with a longer follow-up period.

## 5. Conclusions

This study does not aim to demonstrate the prognostic value of the scintigraphic protocol described, given the strong lack of follow-up data. However, it is hypothesized that more comprehensive data collection would confirm the prognostic role of ^123^I-MIBG scintigraphy and its clinical utility. The analysis supports the objective of stratifying arrhythmic risk in patients with previous myocarditis, using an appropriate statistical model to estimate individual susceptibility to malignant arrhythmic events. Expanding the sample size and ensuring rigorous periodic monitoring of arrhythmic activity could validate the predictive algorithm described.

## Figures and Tables

**Table 1:** Baseline clinical characteristics of study subjects.

Variable	Number of Subjects with Data	Media	Std Deviation	Min	25%	50% (Median)	75%	Max
Age	50	50.96	16.46	15	41	54	63.75	81
EF LV MRI	50	55.60	9.27	26	51	56	61	73
LVEDV MRI	49	164.37	40.06	70	144	161	185	255
RV MRI	50	56.34	7.72	29	52	56	60.75	73
RVEDV MRI	49	175.90	143.88	68	125	157	184	1114
N of LGE segments	50	2.34	1.57	0	1	2	3	8

EF = ejection fraction; LV = left ventricle; RV = right ventricle; EDV = end diastolic volume; MRI = magnetic resonance imaging.

**Table 2:** Population MIBG Data.

Age	Sex	Early_HM_Ratio	Late_HM_Ratio	WOR	Valued_Risk_Level
24	M	2.13	2.21	21.70	0
36	M	1.78	1.87	23.74	0
37	M	1.76	1.84	23.60	0
41	M	1.61	1.83	11.1	0
42	M	1.51	1.74	10.45	0
43	M	1.82	2.21	12.83	0
43	M	1.75	1.94	12.19	0
49	M	1.78	1.9	23.35	0
49	M	1.96	2.06	32.01	0
53	M	1.74	1.68	33.10	0
54	M	1.77	1.92	20.82	0
57	F	1.35	1.35	28.94	1
64	M	1.96	2.07	25.48	0
71	M	1.95	1.89	26.32	0
15	M	2.06	2.16	29.25	0
34	M	1.98	2.32	11.76	0
54	M	1.56	1.63	18.82	0
57	M	1.89	2.11	17.42	0
71	F	2.01	1.98	29.20	0
58	M	1.51	1.66	5.62	0
55	M	1.65	1.74	19.92	0
23	M	1.67	1.75	19.43	0
61	M	1.72	1.57	8.70	0
81	F	1.79	1.58	45.46	1
49	M	2.07	2.66	18.25	0
54	M	1.7	1.82	14.21	0
43	M	1.78	1.86	24	0
76	F	1.42	1.39	27.41	1
75	F	1.93	1.75	38.36	0
67	M	1.94	1.81	32.13	0
41	M	1.78	1.88	23.98	0
45	M	1.85	1.87	18.61	0
39	M	1.58	1.57	58.85	0
70	F	1.91	1.98	11.34	0
66	M	1.76	1.74	33.51	0
19	M	1.65	1.87	11.53	0
71	M	1.69	1.60	35.43	0
37	M	1.95	2.01	27.67	0
28	M	1.81	2.05	8.49	0
64	M	2.28	2.77	23.74	0
63	M	1.76	1.47	49.88	1
68	M	1.84	1.89	11.99	0
63	M	1.83	1.95	5.5	0
58	F	2.01	1.93	28.31	0
47	M	1.58	1.66	14.76	0
20	M	1.67	1.82	10	0
60	M	1.72	1.85	17.59	0
68	M	1.95	1.95	21.56	0
58	F	1.82	1.72	31.08	0
27	F	1.99	2.08	12.42	0

HM = heart-to-mediastinum; WOR = myocardial washout rate.

## Data Availability

The original contributions presented in this study are included in the article. Further inquiries can be directed to the corresponding author.

## References

[1] Brociek E., Tymińska A., Giordani A.S., Caforio A.L.P., Wojnicz R., Grabowski M., Ozierański K. (2023). Myocarditis: Etiology, pathogenesis, and their implications in clinical practice. Biology.

[2] Zhang D.Y., Anderson A.S. (2014). The sympathetic nervous system and heart failure. Cardiol. Clin..

[3] Chirumamilla A., Travin M.I. (2011). Cardiac applications of ^123^I-mIBG imaging. Semin. Nucl. Med..

[4] Casáns-Tormo I., Jiménez-Heffernan A., Pubul-Núñez V., Ruano-Pérez R. (2019). Cardiac sympathetic innervation scintigraphy with ^123^I-meta-iodobenzylguanidine. Basis, protocols and clinical applications in Cardiology. Rev. Esp. Med. Nucl. Imagen Mol. (Engl. Ed.).

[5] Marcassa C. (2021). MIBG and imaging of cardiac adrenergic system: From heart failure to ventricular arrhythmias and atrial fibrillation, through cardiac asynchrony. What else?. J. Nucl. Cardiol..

[6] Verschure D.O., van Eck-Smit B.L.F., Somsen G.A., Knol R.J.J., Verberne H.J. (2016). Cardiac sympathetic activity in chronic heart failure: Cardiac ^123^I-mIBG scintigraphy to improve patient selection for ICD implantation. Neth. Heart J..

[7] Zhou W., Chen J. (2013). I-123 metaiodobenzylguanidine imaging for predicting ventricular arrhythmia in heart failure patients. J. Biomed. Res..

[8] Perkan A., Di Lenarda A., Sinagra G. (2002). Cardiomiopatia dilatativa: Quando è indicata e cosa chiedere alla biopsia endomiocardica. Arch. G. Ital. Cardiol..

[9] Kramer C.M., Barkhausen J., Bucciarelli-Ducci C., Flamm S.D., Kim R.J., Nagel E. (2020). Standardized cardiovascular magnetic resonance imaging (CMR) protocols: 2020 update. J. Cardiovasc. Magn. Reson..

[10] Messroghli D.R., Moon J.C., Ferreira V.M., Grosse-Wortmann L., He T., Kellman P., Mascherbauer J., Nezafat R., Salerno M., Schelbert E.B. (2017). Clinical recommendations for cardiovascular magnetic resonance mapping of T1, T2, T2* and extracellular volume: A consensus statement by the Society for Cardiovascular Magnetic Resonance (SCMR) endorsed by the European Association for Cardiovascular Imaging (EACVI). J. Cardiovasc. Magn. Reson..

[11] Luetkens J.A., Faron A., Isaak A., Dabir D., Kuetting D., Feisst A., Schmeel F.C., Sprinkart A.M., Thomas D. (2019). Comparison of original and 2018 lake louise criteria for diagnosis of acute myocarditis: Results of a validation cohort. Radiol. Cardiothorac. Imaging.

[12] Semenzato L., Le Vu S., Botton J., Bertrand M., Jabagi M.J., Drouin J., Cuenot F., Zores F., Dray-Spira R., Weill A. (2024). Long-Term Prognosis of Patients with Myocarditis Attributed to COVID-19 mRNA Vaccination, SARS-CoV-2 Infection, or Conventional Etiologies. JAMA.

[13] Haaf P., Garg P., Messroghli D.R., Broadbent D.A., Greenwood J.P., Plein S. (2016). Cardiac T1 Mapping and Extracellular Volume (ECV) in clinical practice: A comprehensive review. J. Cardiovasc. Magn. Reson..

[14] Yamamoto H., Yamada T., Tamaki S., Morita T., Furukawa Y., Iwasaki Y., Kawasaki M., Kikuchi A., Kondo T., Ozaki T. (2019). Prediction of sudden cardiac death in patients with chronic heart failure by regional washout rate in cardiac MIBG SPECT imaging. J. Nucl. Cardiol..

[15] Harmon K.G., Asif I.M., Maleszewski J.J., Owens D.S., Prutkin J.M., Salerno J.C., Zigman M.L., Ellenbogen R., Rao A.L., Ackerman M.J. (2016). Incidence and etiology of sudden cardiac arrest and death in high school athletes in the united states. Mayo Clin. Proc..

[16] Maron B.J., Udelson J.E., Bonow R.O., Nishimura R.A., Ackerman M.J., Estes N.M., Cooper L.T., Link M.S., Maron M.S. (2015). Eligibility and disqualification recommendations for competitive athletes with cardiovascular abnormalities: Task force 3: Hypertrophic cardiomyopathy, arrhythmogenic right ventricular cardiomyopathy and other cardiomyopathies, and myocarditis: A scientific statement from the american heart association and american college of cardiology. J. Am. Coll. Cardiol..

[17] Cooper L.T. (2020). Ventricular arrhythmias and sudden cardiac death in lymphocytic myocarditis. J. Am. Coll. Cardiol..

[18] Lo Monaco M., Stankowski K., Figliozzi S., Nicoli F., Scialò V., Gad A., Lisi C., Marchini F., Dellino C.M., Mollace R. (2024). Multiparametric mapping via cardiovascular magnetic resonance in the risk stratification of ventricular arrhythmias and sudden cardiac death. Medicina.

[19] Baksi A.J., Kanaganayagam G.S., Prasad S.K. (2015). Arrhythmias in viral myocarditis and pericarditis. Card. Electrophysiol. Clin..

[20] De Vincentis G., Frantellizzi V., Fedele F., Farcomeni A., Scarparo P., Salvi N., Fegatelli D.A., Mancone M., Verschure D.O., Verberne H.J. (2019). Role of cardiac ^123^I-mIBG imaging in predicting arrhythmic events in stable chronic heart failure patients with an ICD. J. Nucl. Cardiol..

[21] Jacobson A.F., Senior R., Cerqueira M.D., Wong N.D., Thomas G.S., Lopez V.A., Agostini D., Weiland F., Chandna H., Narula J. (2010). Myocardial iodine-^123^ meta-iodobenzylguanidine imaging and cardiac events in heart failure. Results of the prospective ADMIRE-HF (AdreView Myocardial Imaging for Risk Evaluation in Heart Failure) study. J. Am. Coll. Cardiol..

[22] Narula J., Gerson M., Thomas G.S., Cerqueira M.D., Jacobson A.F. (2015). ^123^I-MIBG Imaging for Prediction of Mortality and Potentially Fatal Events in Heart Failure: The ADMIRE-HFX Study. J. Nucl. Med..

